# Differentiation of the frog sculpin *Myoxocephalus stelleri* Tilesius, 1811 (Actinopterygii, Cottidae) based on mtDNA and karyotype analyses

**DOI:** 10.3897/CompCytogen.v15.i2.63207

**Published:** 2021-06-14

**Authors:** Irina N. Moreva, Olga A. Radchenko, Anna V. Petrovskaya

**Affiliations:** 1 A.V. Zhirmunsky National Scientific Center of Marine Biology, Far Eastern Branch, Russian Academy of Sciences, 690041, Vladivostok, ul. Palchevskogo, 17, Russia Russian Academy of Sciences Vladivostok Russia; 2 Institute of Biological Problems of the North, Far Eastern Branch, Russian Academy of Sciences, 685000, Magadan, ul. Portovaya, 18, Russia Russian Academy of Sciences Magadan Russia

**Keywords:** 16S rRNA, *COI*, cytochrome *b*, cytotype, haplotype, Myoxocephalinae, Robertsonian polymorphism, Sea of Japan, Sea of Okhotsk

## Abstract

A molecular genetic and karyological study of the frog sculpin *Myoxocephalus stelleri* Tilesius, 1811 was carried out on an extensive sample from a large area of the species’ range. A total of 42 specimens was sampled from the Sea of Japan, Sea of Okhotsk, and coastal waters off the southern Kuril Islands, which makes this sampling scheme the most comprehensive to date. The level of mtDNA polymorphism was found to be low. The haplotypes of the species formed three phylogenetic groups. The unique *M. stelleri* haplotype from the coast of Shikotan Island linked all the studied groups, indicating that it is likely ancestral. Robertsonian polymorphism was identified in the species. In all five cytotypes (I – 2n = 44, II – 2n = 43, III – 2n = 42, IV – 2n = 41, V – 2n = 40; NF = 44+2) were identified, all of which were present in the Sea of Japan. Only one (cytotype I) was found in the Sea of Okhotsk, which is probably the closest to the ancestral karyotype. The significant chromosomal polymorphism and the presence of common haplotypes in the studied samples indicate their recent origin from a common ancestor and/or relatively recent contacts within the range. The discrepancies between mtDNA and karyotypes in assigning the ancestral *M. stelleri* to the coastal waters off Shikotan Island (southern Kuril Islands) and the Sea of Okhotsk, respectively, can be explained by the different inheritance mechanisms and the rates of evolution of molecular genetic and karyological traits.

## Introduction

The genus *Myoxocephalus* Tilesius, 1811 is a large, taxonomically complex group of sculpins of the subfamily Myoxocephalinae (family Cottidae) (Neelov 1979). The modern world catalog (Fricke et al. 2020) lists 14 species and subspecies of this genus, which inhabit coastal and shelf zones of the Atlantic, Arctic, and Pacific Oceans (Neelov 1979; Parin et al. 2014; Mecklenburg et al. 2016). The northern Pacific Ocean is considered the center of diversification of this genus (Nazarkin 2000). The frog sculpin *Myoxocephalus stelleri* Tilesius, 1811 is one of the most common species of the genus, found across a wide geographic range from the Sea of Japan to the Gulf of Alaska (Mecklenburg et al. 2002). *M. stelleri* is characterized by significant morphological and ecological plasticity, as it tolerates substantial fluctuations in water temperature and salinity, being found even in river estuaries (Neelov 1979; Sokolovsky et al. 2011). Similar species, including *M. decastrensis* (Kner, 1865) and *M. raninus* Jordan et Starks, 1904 (Parin et al. 2014; Fricke et al. 2020), which are now considered to be junior synonyms of *M. stelleri*, have been described from various parts of the *M. stelleri* range.

Cytogenetic and genetic studies of *M. stelleri* have been described in two publications (Miller 2000; Podlesnykh and Moreva 2014). The only molecular genetic study thus far was based on a small number of specimens from Amur Bay, Sea of Japan, and Odyan Bay, Sea of Okhotsk (Podlesnykh and Moreva 2014). The study highlighted the limitations of using the short fragment of the *CO1* mitochondrial gene (525 bp) when constructing an adequate system of the *Myoxocephalus* species due to the lack of informative characters. The frog sculpin karyotype was first described from Amur Bay, Sea of Japan (Miller 2000). Unlike other *Myoxocephalus* species from the Sea of Japan and Sea of Okhotsk (Radchenko et al. 2020), *M. stelleri* exhibits chromosomal variation. Karyological analysis of morphologically indistinguishable individuals from Odyan Bay and Amur Bay revealed their differences not only in the diploid numbers (2n) (2n = 44 vs. 2n = 40; fundamental numbers (NF) 44 + 2), but also in the number and localization of active nucleolar organizers (NORs) (Podlesnykh and Moreva 2014). This led the authors to suggest revising the species affiliation of *M. stelleri* from the Sea of Japan (Podlesnykh and Moreva 2014). However, the same study showed that the *M. stelleri* clade is a combination of haplotypes of specimens not only from different geographic localities, but also with different karyotypes. Hence, the discrepancy between the genetic and cytogenetic results, along with the small geographic area of sampling and the limited resolution of the short *CO1* fragment, warrant further studies.

We conducted a study to determine the level of genetic and karyological differentiation within and between *M. stelleri* from the Sea of Japan, Sea of Okhotsk, and the southern Kuril Islands. Using this karyological (N = 42) and molecular genetic data (N = 34), we also aimed to find the centers of species diversification. Our extensive sample included individuals of *M. stelleri* captured from waters near the site of the original species description – the estuary of the Bolshaya Zapadnaya Kamchatka River (Tilesius 1811) – and also from Chikhachev Bay (De Kastri), which is the type locality of *M. decastrensis* (Fricke et al. 2020). The results obtained allow assessment of the geographical variation of *M. stelleri* and identification of its probable causes.

## Materials and methods

### Sample collection

**Figure 1.**
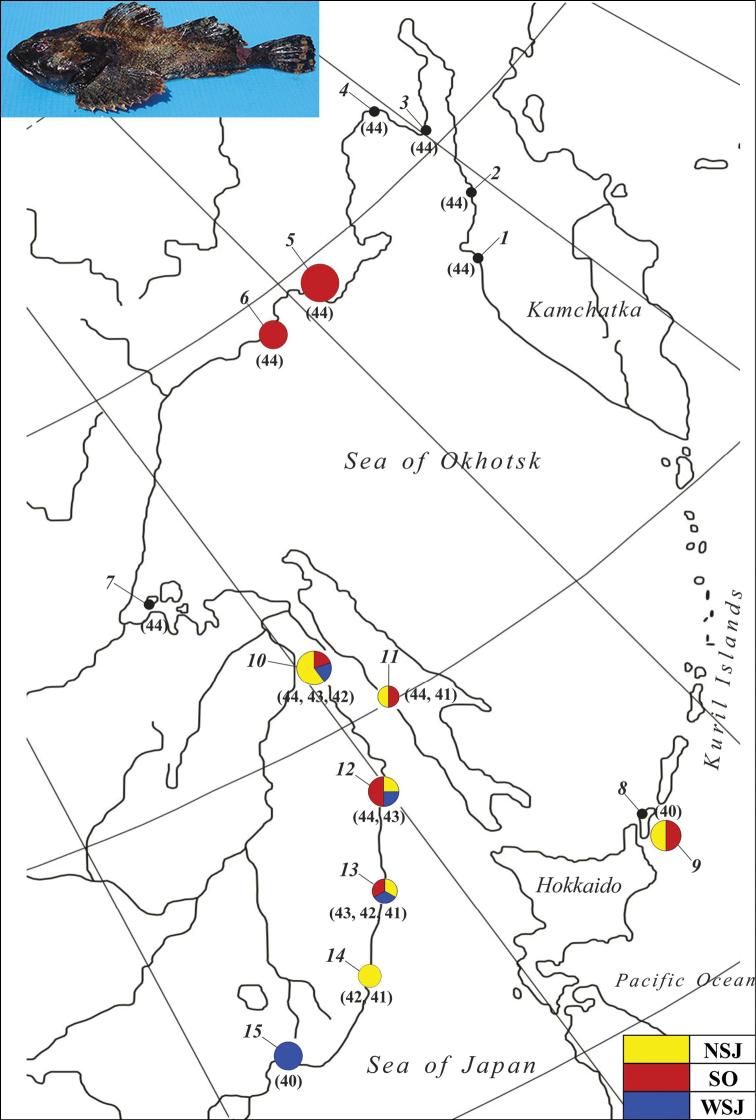
The sampling localities, distribution of haplotype groups (colored circles), and diploid numbers (2n in parentheses) in the cytotypes of *M. stelleri*. The locality numbers are listed in Table 1.

Fig. 1 and Table 1 show the sampling sites and number of specimens examined. Fish were collected from 2016 to 2019, at 20 localities in coastal waters of the Sea of Japan (7 localities), Sea of Okhotsk (10 localities), and off the southern Kuril Islands (3 localities). Species were identified by morphological traits (Neelov 1979). The Sea of Japan is divided into the northern and western parts according to Appendix to “Nekton of the Northwestern part of Japan (East) Sea” (Shuntov et al. 2004). The fish specimens are stored at the Ichthyological laboratory collection of the Institute of Biological Problems of the North, Far Eastern Branch, Russian Academy of Sciences, Russia (voucher numbers are listed in Table 1).

**Table 1.** Specimens of *M. stelleri* and outgroup species examined (*specimens whose mtDNA was not studied).

Species (genetic voucher)	Locality	GenBank accession numbers		
		*COI*	Cyt *b*	16S rRNA
Sea of Japan				
*M. stelleri* (1754)	Peter the Great Bay, Vostok Bay (15)	KY062754	MH595735	KY062665
*M. stelleri* (1755)	Peter the Great Bay, Vostok Bay (15)	MN115304	MN115340	MN097160
*M. stelleri* (1756)	Peter the Great Bay, Vostok Bay (15)	MN115305	MN115341	MN097161
*M. stelleri* (2097)	Peter the Great Bay, Russky Island (15)	MN115306	MN115342	MN097162
*M. stelleri* (1991)	Olga Bay (14)	MN115311	MN115347	MN097167
*M. stelleri* (2081)	Olga Bay (14)	MN115312	MN115348	MN097168
*M. stelleri* (2044)	Zolotaya Bay (12)	MN115313	MN115349	MN097169
*M. stelleri* (2083)	Zolotaya Bay (12)	MT258533	MT253729	MT251919
*M. stelleri* (2149)	Zolotaya Bay (12)	MN115314	MN115350	MN097170
*M. stelleri* (2150)	Zolotaya Bay (12)	MN115315	MN115351	MN097171
*M. stelleri* (2047)	Dzhigit Bay (13)	MN115316	MN115352	MN097172
*M. stelleri* (2082)	Dzhigit Bay (13)	MN115317	MN115353	MN097173
*M. stelleri* (2148)	Dzhigit Bay (13)	MN115318	MN115354	MN097174
*M. stelleri* (1985)	Aleksandrovsky Bay (11)	MN115319	MN115355	MN097175
*M. stelleri* (2084)	Aleksandrovsky Bay (11)	MN115320	MN115356	MN097176
*M. stelleri* (2151)	Chikhachev Bay (10)	MN115321	MN115357	MN097177
*M. stelleri* (2152)	Chikhachev Bay (10)	MN115322	MN115358	MN097178
*M. stelleri* (2049)	Chikhachev Bay (10)	MN115323	MN115359	MN097179
*M. stelleri* (1973)	Chikhachev Bay (10)	MN115324	MN115360	MN097180
*M. stelleri* (2153)	Chikhachev Bay (10)	MN115325	MN115361	MN097181
Pacific Ocean, Shikotan Island				
*M. stelleri* (1736)	Gorobets Bay (9)	MN115307	MN115343	MN097163
*M. stelleri* (1737)	Krabovaya Bay (9)	MN115308	MN115344	MN097164
*M. stelleri* (1739)	Otradnaya Bay (9)	MN115309	MN115345	MN097165
*M. stelleri* (1740)	Otradnaya Bay (9)	MN115310	MN115346	MN097166
Sea of Okhotsk				
M. stelleri*	Kunashir Island, Pervukhin Bay (8)	–	–	–
*M. stelleri* (1748)	Taui Bay, Uta River estuary (6)	MN115326	MN115362	MN097182
*M. stelleri* (1749)	Taui Bay, Shestakov Bay (6)	MN115327	MN115363	MN097183
*M. stelleri* (1778)	Taui Bay, Shestakov Bay (6)	MN115328	MN115364	MN097184
*M. stelleri* (1780)	Taui Bay, Shestakov Bay (6)	MN115329	MN115365	MN097185
*M. stelleri* (2077)	Taui Bay, Odyan Bay (5)	MN115330	MN115366	MN097186
*M. stelleri* (2078)	Taui Bay, Odyan Bay (5)	MN115331	MN115367	MN097187
*M. stelleri* (2133)	Taui Bay, Odyan Bay (5)	MN115332	MN115368	MN097188
*M. stelleri* (2134)	Taui Bay, Odyan Bay (5)	MN115333	MN115369	MN097189
*M. stelleri* (2131)	Taui Bay, Nedorazumeniya Island (5)	MN115334	MN115370	MN097190
*M. stelleri* (2132)	Taui Bay, Nedorazumeniya Island (5)	MN115335	MN115371	MN097191
M. stelleri*	Feklistov Island (7)	–	–	–
	Feklistov Island (7)	–	–	–
	Shelikhov Bay, Gizhigin Bay (4)	–	–	–
	Shelikhov Bay, Gizhigin Bay (4)	–	–	–
	Shelikhov Bay, Penzhina Bay (3)	–	–	–
	Western Kamchatka, Kvachin Bay (2)	–	–	–
	Western Kamchatka, Kvachin Bay (2)	–	–	–
	Western Kamchatka, Pichgygyn Bay (1)	–	–	–
Outgroups				
*M. jaok*	Sea of Japan	MN115336	MN115372	MN097192
*M. brandtii*	Shikotan Island	MN115337	MN115373	MN097193
*M. polyacanthocephalus*	Shikotan Island	MN115338	MN115374	MN097194
*M. ochotensis*	Sea of Okhotsk	MN115339	MN115375	MN097195

### Ethics statements

This study utilizes samples collected with all applicable international, national and/or institutional guidelines for sampling, care and experimental use of organisms. The fishes studied here are not included in the IUCN Red List of Threatened Species, nor are they are listed as endangered, vulnerable, rare, or protected species in the Russian Federation. The sampling points are located beyond any protected areas.

### DNA analysis

We obtained sequences for three mtDNA markers: *COI*, cytochrome *b*, and 16S rRNA. Total DNA was extracted from muscle tissues by standard phenol extraction (Maniatis et al. 1982) following tissue lysing with 1% SDS using Proteinase K (0.2 mg/mL). The following oligonucleotide primers were used to amplify and sequence the DNA markers: for *COI*, F-33 TCACAAAGACATTGGCACCCTA and R-1421 TTCACGTTTAGCAGCGAATGCTT (Moreva et al. 2017); for cytochrome *b*, L14795 CAATGGCAAGCCTACGAAAA and H15844 AGCTACTAGTGCATGACCATC (Radchenko 2005); for 16S rRNA, L2510 CGCCTGTTTATCAAAAACAT and H3080 CCGGTCTGAACTCAGATCACGT (Meyer 1993).

DNA sequences were aligned in Clustal W (MEGA version X: Kumar et al. 2018) with default settings, and manually edited after visual inspection. To identify haplotypes and their relationships, a median network was built in SplitsTree4 v4.12.3 (Huson and Bryant 2006) using the median-joining method (Bandelt et al. 1999). Genetic distances (d) between haplotypes were calculated using *p*-distances in MEGA X. DNA sequences were first analyzed for each gene independently, then a concatenated matrix was created in which different sets of partition scenarios were investigated. For each gene, we used MEGA X under Bayesian Information Criterion (BIC) and Akaike Information Criterion (AIC) to select the optimal models of nucleotide substitutions. Bayesian Inference trees were constructed using MrBayes v3.2.1 (Ronquist et al. 2012), with the prior set to fit the evolutionary models suggested by MEGA X but allowing the parameters to be recalculated during the run. The Markov Chain Monte Carlo process was set for four chains to be run simultaneously for 1,000,000 generations, with trees sampled every 100 generations. Out of the total 10,001 obtained trees, the first 1,001 with unstable parameters of the models of nucleotide substitutions were rejected. The Bayesian analysis dynamics were controlled in Tracer v1.5 (http://tree.bio.ed.ac.uk/software/tracer/). Nodes with posterior probabilities ≥0.95 were accepted as statistically significant (Leache and Reeder 2002). We used the following four congeneric species of the subfamily Myoxocephalinae as outgroups: *Myoxocephalus jaok* (Cuvier, 1829), *M. brandtii* (Steindachner, 1867), *M. polyacanthocephalus* (Pallas, 1814), and *M. ochotensis* Schmidt, 1929.

### Karyological analysis

Chromosomes were prepared by the air-drying technique (Kligerman and Bloom 1977). Slides were stained with a 4% azure eosin solution (Giemsa, Merck, Germany) in 0,067 M Sorenson’s buffer contains 4.53 g KH_2_PO_4_ and 4.72 g Na_2_HPO_4_ per liter of distilled water. Metaphase plates were examined under a Leica microscope. The best metaphase plates were photographed using an AxioCam HR CCD camera with the AXIOVISION software (Carl Zeiss MicroImaging GmbH, Germany). Microscopy and imaging were carried out at the Far Eastern Center of Electron and Light Microscopy, A.V. Zhirmunsky National Scientific Center of Marine Biology (NSCMB), FEB RAS, Vladivostok.

Chromosomes were classified according to the nomenclature of Levan et al. (1964). Metacentric chromosomes with equal arms and submetacentric chromosomes with unequal arms were referred to as bi-armed chromosomes; subtelocentric chromosomes with a very short second arm and acrocentric chromosomes with an invisible second arm were referred to as uni-armed chromosomes. Submeta-subtelocentrics were also distinguished in the cytotypes. The chromosomes of this pair are morphologically highly variable. In different cells, they may look like submetacentrics or subtelocentrics. For this reason, the number of chromosome arms (NF) is indicated with both possible variants: 44 + 2.

## Results

### DNA analysis

For *M. stelleri*, the length of the partial *COI* was 1,009 base pairs (bp), including 16 variable sites, 8 parsimony informative sites, and 5 non-synonymous substitutions. The length of the partial cytochrome *b* was 747 bp, including 16 variable sites, 10 parsimony informative sites, and 1 non-synonymous substitution. The length of the partial 16S rRNA was 600 bp, including 1 variable site and 1 parsimony informative site. All sequence data are deposited in GenBank/NCBI (www.ncbi.nlm.nih.gov) (for accession numbers, see Table 1). Based on the parsimony informative sites, 13 haplotypes were found (Table 2).

**Table 2.** Haplotypes of *M. stelleri* (nucleotide substitutions indicating phylogenetic groups are highlighted in bold).

Haplotype	Parsimony informative nucleotide sites			Locality (see sampling site nos. in Table 1 and Fig. 1)
	*COI*	Cyt*b*	16S rRNA	
1a	C**TA**AT**T**TC	T**CT**T**G**CTTC**T**	**A**	9
2a	C**TA**AC**T**TC	T**CT**T**G**CTTC**T**	**A**	9, 10, 12, 13, 14
3a	C**TA**AC**T**TC	A**CT**T**G**CTTC**T**	**A**	11, 14
1b	C**CA**AT**C**TC	T**CT**T**A**CTTC**T**	**G**	9
2b	T**CA**AT**C**TC	T**CT**C**A**TTTC**T**	**G**	5, 6, 10
3b	C**CA**AT**C**TC	T**CT**T**A**TCTC**T**	**G**	5, 9, 11, 12, 13
4b	C**CA**AT**C**TC	T**CT**T**A**TCCC**T**	**G**	5, 6
5b	C**TA**AT**C**TC	T**CT**T**A**TCTC**T**	**G**	12
6b	C**CA**AT**C**TC	A**CT**T**A**TCTC**T**	**G**	5
1c	C**CG**AT**C**TC	T**TC**T**G**CTTC**C**	**G**	12
2c	C**CG**AT**C**CC	T**TC**T**G**CTTC**C**	**G**	13, 15
3c	C**CG**AT**C**CT	T**TC**T**G**CTTC**C**	**G**	10, 15
4c	C**CG**GT**C**CC	T**TC**T**G**CTTT**C**	**G**	15

**Figure 2.**
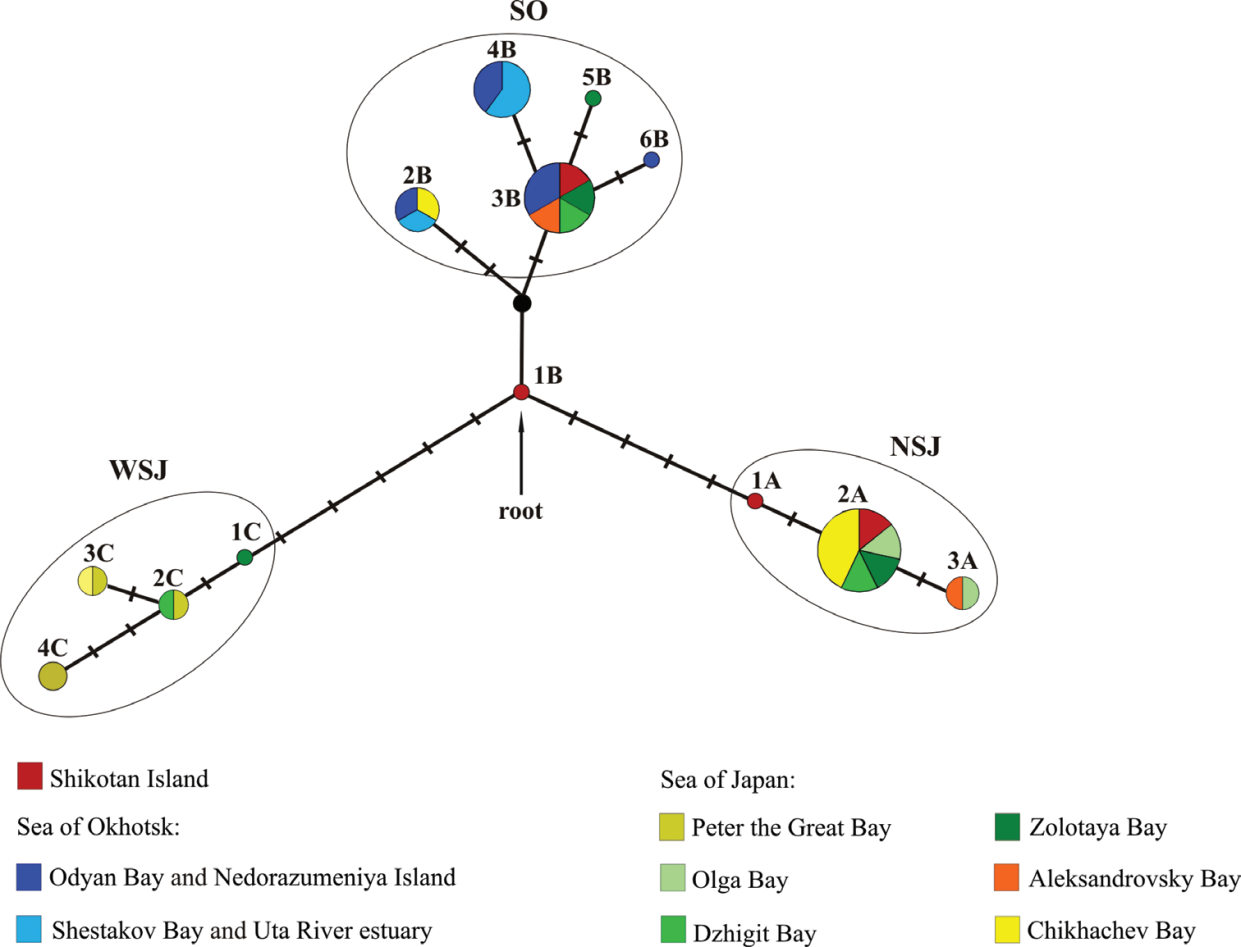
A median network of the *M. stelleri* haplotypes based on mtDNA sequences. Each haplotype is represented by a circle; its size corresponds to the number of individuals with this haplotype. Black circle indicates a hypothetical (unsampled) haplotype. Colors designate the geographic distribution of haplotypes. Markings on the branches are nucleotide substitutions. Ellipses are the haplotype groups: NSJ (Northern Sea of Japan group); SO (Sea of Okhotsk group); WSJ (Western Sea of Japan group).

Haplotype polymorphism is determined by single-nucleotide mutations. The minimum difference (one substitution) was found between haplotypes 3b vs. 4b, 3b vs. 5b, 1c vs. 2c, and 2c vs. 3c; the maximum difference (14 substitutions) was found between haplotypes 2a vs. 4c and 3a vs. 4c (Fig. 2). Among the identified haplotypes, five were unique (1a, 1b, 5b, 6b, and 1c) with a total frequency of 14.7%, and three were common and widespread (2a, 3b, and 4b) with a total frequency of 53%.

The haplotype network for *M. stelleri* is a star-shaped structure with the central haplotype (1b) from Shikotan Island (Fig. 2). There were three haplogroups, each formed largely by the haplotypes from the same geographic area. Group NSJ (the Northern Sea of Japan group) includes three haplotypes, of which 2a is the most common. Haplotypes of this group are found in the northern Sea of Japan and in the coastal waters off the southern Kuril Islands. Group SO (the Sea of Okhotsk group) has six haplotypes, of which 3b is the most common. This group includes all specimens from the Sea of Okhotsk, as well as individuals from the northern Sea of Japan and the southern Kuril Islands. Group WSJ (the Western Sea of Japan group) has four haplotypes and includes all specimens from the western Sea of Japan and the northern part of the Sea of Japan.

We identified the nucleotide substitutions that distinguished the haplogroups. In Group NSJ, there is one substitution in the 16S rRNA gene (G → А at position 313) and one in *COI* (C → Т at position 495). In Group SO, there is only one substitution in the cytochrome *b* gene (А → G at position 195). In Group WSJ, there are three substitutions: one in the *COI* gene (G → А at position 318) and two in cytochrome *b* (Т → C at position 36, C → Т at positions 75 and 639; nucleotide positions are according to our matrix).

**Figure 3.**
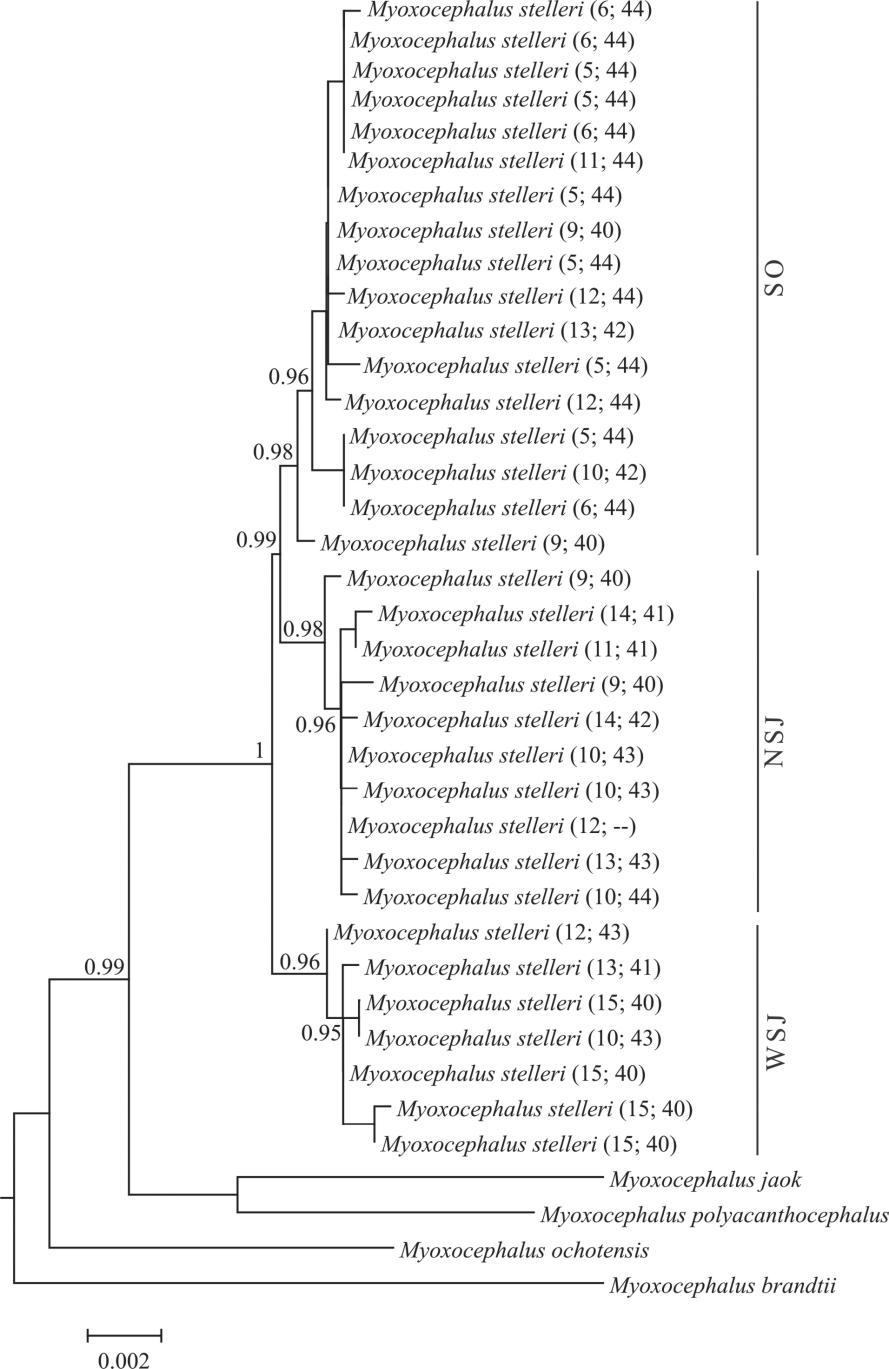
Bayesian Inference tree based on mtDNA marker sequences. The numerals at nodes are posterior probability values. The numerals in parentheses are the locality numbers and diploid numbers (2n) as in Fig. 1.

The Bayesian tree (Fig. 3) shows three major clades that are congruent with the three haplogroups described above (NSJ, SO, and WSJ). Group WSJ is basal, while groups NSJ and SO are sister clades. The main nodes are supported by significant posterior probability values (≥0.95).

### Karyological analysis

**Figure 4.**
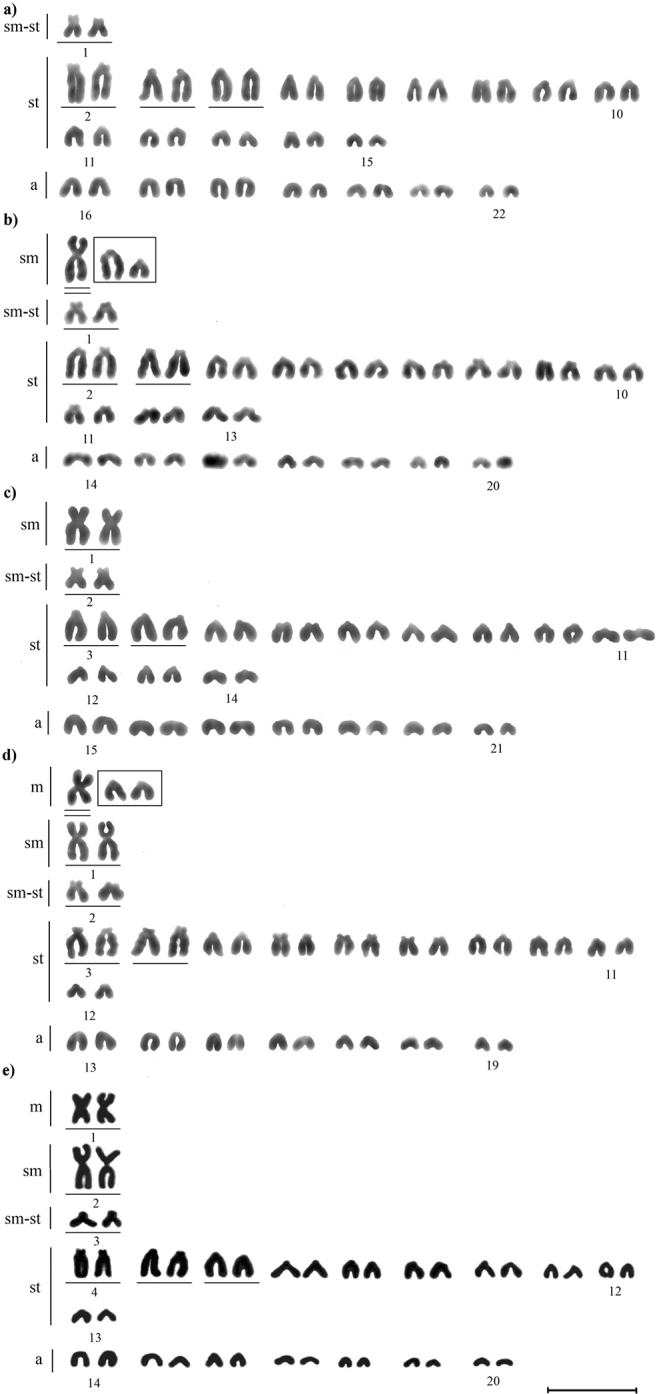
Karyograms of *M. stelleri*: (**a**) cytotype I, 2n = 44; (**b**) cytotype II, 2n = 43; (**c**) cytotype III, 2n = 42; (**d**) cytotype IV, 2n = 41; (**e**) cytotype V, 2n = 40 (according to Moreva and Borisenko 2017); NF = 44+2. The letter designations are as follows: m, metacentric; sm, submetacentric; sm-st, submeta-subtelocentric; st, subtelocentric; a, acrocentric chromosomes; the non-homologous subtelocentric chromosomes are boxed; the marker chromosomes are underlined once; the chromosomes similar in size to the marker metacentrics and submetacentrics are underlined twice. Scale bar: 10 μm.

Five cytotypes were found in *M. stelleri*. Their major characteristics were described based on the analysis of 1,520 metaphase plates (Table 3 and Fig. 4).

**Table 3.** The cytotypes of *M. stelleri* and their geographic distribution (juv = juvenile individuals).

No. of cytotype	Number / sex of individuals	Number of metaphase plates	Locality (see sampling sites nos. in Table 1 and Fig. 1)	Region
I	22	551	1–7, 10–12	Sea of Okhotsk; northern Sea of Japan
	(10♀, 10♂, 2 juv)			
II	5	271	10, 12, 13	Northern Sea of Japan
	(1 ♀, 2 ♂, 2 juv)			
III	3	130	10, 13, 14	Northern Sea of Japan
	(1 ♀, 1 ♂, 1 juv)			
IV	3	148	11, 13, 14	Northern Sea of Japan
	(2 ♂, 1 juv)			
V	9	420	8, 9, 15	Western Sea of Japan; coastal waters off the southern Kuril Islands
	(3 ♀, 5 ♂, 1 juv)			

Cytotype I: 2n = 44 chromosomes (Fig. 4А), with the first two chromosomes identified as submeta-subtelocentrics; the row of uni-armed chromosomes contains 28 subtelocentrics (pairs 2 to 15) and 14 acrocentrics (pairs 16 to 22).

Cytotype II: 2n = 43 (Fig. 4b), includes one odd submetacentric, two submeta-subtelocentrics (pair 1), 24 subtelocentrics (pairs 2 to 13), two non-homologous subtelocentrics (box), and 14 acrocentrics (pairs 14 to 20).

Cytotype III: 2n = 42 (Fig. 4c), includes two submetacentrics (pair 1), two submeta-subtelocentrics (pair 2), 24 subtelocentrics (pairs 3 to 14), and 14 acrocentrics (pairs 14 to 20).

Cytotype IV: 2n = 41 (Fig. 4d), includes one odd metacentric, two submetacentrics (pair 1), two submeta-subtelocentrics (pair 2), 20 subtelocentrics (pairs 3 to 12), two possibly non-homologous subtelocentrics (box), and 14 acrocentrics (pairs 13 to 19).

Cytotype V: 2n = 40 (Fig. 4e), includes two metacentrics (pair 1), two submetacentrics (pair 2), two submeta-subtelocentrics (pair 3), 20 subtelocentrics (pairs 4 to 13), and 14 acrocentrics (pairs 14 to 20).

All cytotypes have an equivalent number of chromosome arms: 44 + 2 (Fig. 4).

The markers of cytotypes III–V are two submetacentrics (Fig. 4c: pair 1; Fig. 4d: pair 1; Fig. 4e: pair 2), which are the largest of all chromosomes in these cytotypes; the markers of cytotype V are two metacentrics (Fig. 4e: pair 1), which are similar in size to four large subtelocentrics (Fig. 4e: pairs 4 and 5). The odd submetacentric of cytotype II (Fig. 4b) is similar in size to the large marker submetacentrics of cytotypes III–V (Fig. 4c, d: pair 1; Fig. 4e: pair 2). The size of the unpaired metacentric of cytotype IV (Fig. 4d) is close to those of the large marker metacentrics of cytotype V (Fig. 4e: pair 1). Two submeta-subtelocentrics (Fig. 4a, b: pair 1; Fig. 4c, d: pair 2; Fig. 4e: pair 3) and four large subtelocentrics (Fig. 4a, b: pairs 2 and 3; Fig. 4c, d: pairs 3 and 4; Fig. 4e: pairs 4 and 5) are clearly distinguishable in the metaphase plates and are the markers for all cytotypes. Cytotypes I and V have additional markers: two large subtelocentrics (Fig. 4А: pair 4; Fig. 4e: pair 6), which are smaller than the large marker subtelocentrics (Fig. 4А: pairs 2 and 3; Fig. 4e: pairs 4 and 5) but larger than all other uni-armed chromosomes of these cytotypes. The pairs of homologous subtelocentrics and acrocentrics in the presented karyograms are in a row from large to small (Fig. 4).

## Discussion

Our DNA data show that *M. stelleri* is genetically diverse at various levels: within the same locality, between geographically close localities, and between geographically distant areas. Each sample had 2 to 4 haplotypes, some of which were unique and others widespread; however, there were no haplotypes common to all studied samples. Several samples had specific haplotypes, e.g. haplotypes 4b (frequency 14.7%) and 6b were found only in the Sea of Okhotsk, while haplotype 4c (frequency 5.9%) was found only in Peter the Great Bay. Similarly, the unique haplotypes 1a and 1b were found in *M. stelleri* from the coastal waters off the southern Kuril Islands, and haplotypes 1c and 5b were found only in the Zolotaya Bay sample.

The most common haplotype (2a; frequency of 20.6%) was found in *M. stelleri* from the northern Sea of Japan (Chikhachev Bay, Zolotaya Bay, Dzhigit Bay, and Olga Bay) and from the coastal waters off the southern Kuril Islands. Haplotype 3b (17.7%) was distributed wider across the species range, from the southern Kuril Islands and the northern Sea of Japan to the Sea of Okhotsk. The less common haplotypes (3a, 2b, 2c, and 3c) were also found in more than one sample. The common haplotypes from the Sea of Okhotsk, Sea of Japan, and the southern Kuril Islands can be explained either by their origin from a common ancestor followed by dispersal from the same center, or by recent contact in various parts of the species range. A pattern of haplotype distribution with a few haplotypes being very common and others being rare or unique is frequently found in marine fish (Avise 2000).

In general, *M. stelleri* from the northern Sea of Japan exhibits higher genetic variation: eight haplotypes, many of which are shared with other geographic areas, and a low frequency of unique haplotypes. The variation between mtDNA sequences of *M. stelleri* from different localities is low, with the genetic distance between samples being approximately at the same level (Table 4). An exception is the southernmost sample from Peter the Great Bay, which is the most divergent (d = 0.34–0.52%).

**Table 4.** Genetic distances (d) between mtDNA of *M. stelleri* (%).

Localities (see sampling site nos. in Table 1 and Fig. 1)		1	2	3	4	5	6	7	8
1	Odyan Bay + Nedorazumeniya Island (5)								
2	Shestakov Bay + Uta River estuary (6)	0.10							
3	Peter the Great Bay (15)	0.45	0.47						
4	Olga Bay (14)	0.40	0.43	0.52					
5	Dzhigit Bay (13)	0.29	0.31	0.34	0.33				
6	Zolotaya Bay (12)	0.22	0.24	0.36	0.30	0.27			
7	Aleksandrovsky Bay (11)	0.21	0.24	0.46	0.22	0.28	0.23		
8	Chikhachev Bay (10)	0.36	0.35	0.40	0.24	0.30	0.28	0.26	
9	Shikotan Island (9)	0.24	0.26	0.44	0.24	0.28	0.24	0.21	0.26

The mtDNA haplotypes form three haplogroups, congruent with the three clades formed in the phylogenetic tree. These haplotypes belong to different phylogenetic groups: NSJ, SO, and WSJ. There were more differences within the Sea of Japan (groups NSJ and WSJ: d = 0.47%) than between the Sea of Okhotsk and the Sea of Japan (SO and NSJ: d = 0.36%; SO and WSJ: d = 0.42%). The geographic distribution of haplotypes is not uniform (Fig. 1). *M. stelleri* from the Sea of Okhotsk have only SO haplotypes, while haplotypes from all phylogenetic groups were found in the Sea of Japan. For example, in the northern part of the sea, NSJ haplotypes were found at a frequency of 50%, SO haplotypes occurred at a frequency of 31%, and WSJ haplotypes at a frequency of 19%. However, in the western part, only WSJ haplotypes were found. In the Pacific coastal waters off the southern Kuril Islands, NSJ and SO haplotypes were found with equal frequencies.

Two unique haplotypes, 1a and 1b, were found in the coastal waters off Shikotan Island. In the Bayesian tree (Fig. 3), these haplotypes have a basal position in clades NSJ and SO; in the haplotype network (Fig. 2), haplotype 1b has a central position connecting all haplogroups, indicating that it is an ancestral haplotype. Such position of the Shikotan (Pacific) haplotypes probably shows the ancestral role that this population played in the formation of the *M. stelleri* species pattern, as evidenced by the star-shaped network with the central “founder” haplotype. This structure is typical when a population historically experienced an exponential increase in numbers after an earlier reduction in effective population size (bottleneck event) (Avise 2000).

The variation between haplotypes from different parts of the geographic range is most clear between the most distant localities: Shikotan Island vs. the western Sea of Japan, or the Sea of Okhotsk vs. the western Sea of Japan. Similar DNA differentiation has been reported for Cottidae species found in the Sea of Okhotsk and the Sea of Japan, documented both at the subspecies level, e.g. *Megalocottus platycephalus platycephalus* (Pallas, 1814) vs. *M. platycephalus taeniopterus* (Kner, 1868) (Radchenko and Petrovskaya 2019), and at the species level, e.g. species of the genera *Enophrys* Swainson, 1839 and *Porocottus* Gill, 1859 (Moreva et al. 2017, 2019). This trend has also been observed in other fish families: *Lycodes matsubarai* Toyoshima, 1985 (Zoarcidae; Sakuma et al. 2014), *Bothrocara hollandi* (Jordan et Hubbs, 1925) (Zoarcidae; Kodama et al. 2008), and the southern and northern forms of *Tribolodon hakonensis* (Günther, 1877) (Cyprinidae; Ryasanova and Polyakova 2012). In our case, the mtDNA differentiation of *M. stelleri* from the Sea of Okhotsk and the Sea of Japan is not higher than the intraspecific level (d = 0.21–0.47%).

The results of the karyological analysis are consistent with the conclusion drawn from the genetic data: *M. stelleri* is a heterogeneous species (Figs 1, 3). We have shown that *M. stelleri* has a higher chromosomal polymorphism than was previously assumed (Podlesnykh and Moreva 2014). The highest variation was found among individuals sampled from the northern Sea of Japan: 2n = 44 (cytotype I), 2n = 43 (cytotype II), 2n = 42 (cytotype III), and 2n = 41 (cytotype IV). In the western Sea of Japan, the coastal waters off the southern Kuril Islands (cytotype V; 2n = 40), and in the Sea of Okhotsk (cytotype I), individuals are stable in terms of diploid number (Table 3; Figs 1, 4).

All cytotypes of *M. stelleri* differed in the 2n (Fig. 4). Unlike other cytotypes, cytotype I lacks metacentrics and large submetacentrics. Cytotypes II–V contain different numbers of bi-armed chromosomes. All cytotypes differ in the number of subtelocentrics. Despite these differences, there are also traits common to all chromosome sets (Fig. 4). These similarities, as well as NF (44 + 2), suggest that the metacentrics and submetacentrics in cytotypes II–V were formed through Robertsonian fusions. Karyological studies show that Robertsonian translocations are the main mechanism of structural changes in the chromosomes of marine sculpins of the family Cottidae (Vasilyev 1985; Terashima and Ida 1991; Moreva et al. 2017, 2019; Radchenko et al. 2020). Here we report the highest intraspecific variability in 2n (40 to 44) documented to date for this fish group (Arai 2011; Moreva et al. 2017, 2019; Moreva 2020).

The differences in the number of subtelocentrics between the chromosome sets (Fig. 4) indicate that these chromosomes have been involved in the formation of metacentrics and submetacentrics in cytotypes II–V. Cytotypes II–IV lack a pair of large subtelocentrics, which are the additional markers of cytotypes I and V. This fact, along with the size of the non-homologous chromosomes of cytotype II (Fig. 4b, boxed), suggests that the submetacentrics of cytotypes II–IV were formed through the Robertsonian fusion of large subtelocentrics (additional markers of cytotype I) with small subtelocentrics. Other subtelocentrics were involved in the formation of large submetacentrics of cytotype V; these were most likely the medium-sized subtelocentrics of cytotype I. Our data show that the large submetacentrics in cytotypes II–IV and cytotype V formed because of various Robertsonian fusions.

The low level of genetic differentiation in mtDNA between the studied *M. stelleri* (Fig. 3) confirms their close relationship. The karyological analysis suggests that the cytotype I (2n = 44) is close to the ancestral karyotype of the genus *Myoxocephalus* in general (Moreva and Borisenko 2017). Within Group SO (Fig. 3), the frequency of cytotype I was 76%. Among the individuals of this group that had 2n = 44, 77% were from the Sea of Okhotsk and 23% were from the northern Sea of Japan (Aleksandrovsky Bay, Zolotaya Bay). Cytotype I was not found among the individuals from the western Sea of Japan (Fig. 3, Group WSJ), which can be explained by their significant karyological divergence from the individuals from the Sea of Okhotsk and the northern Sea of Japan.

The significant chromosomal variability of individuals from the northern Sea of Japan may indicate their later divergence as compared to individuals from the western part of the sea. The assumption about different divergence times from the Okhotsk Sea is confirmed by the fact that the formation of submetacentrics of similar size in their cytotypes (II–IV and V) occurred because of different Robertsonian translocations. We assume that the polymorphism observed in individuals from the northern Sea of Japan could arise in the relatively recent past in the following way: the chromosomal rearrangement that took place in one or more individuals (Fig. 4b, cytotype II) then became fixed (Fig. 4c, cytotype III) and distributed among *M. stelleri* from the northern Sea of Japan. Cytotype IV (Fig. 4d) from the northern Sea of Japan could be formed because of subsequent hybridization of karyotypically different individuals from the northern and western Sea of Japan. Despite the lack of isolation and their geographic proximity to individuals from the northern Sea of Japan (cytotypes II–IV; Fig. 1), the modern *M. stelleri* from the western part of the sea have a uniform number of chromosomes (cytotype V) (Miller 2000; Podlesnykh and Moreva 2014). This may indicate the lack of secondary contact between *M. stelleri* from the western and northern Sea of Japan at present.

The frog sculpins from the coastal waters off the southern Kuril Islands deserve special attention. In the geological history of the basins of the Far Eastern seas, several long-lasting barriers existed during the regressions of the level of the World Ocean, separating the unified area of the species. One of them, extending along Sakhalin/Hokkaido/Honshu, kept the Japanese Sea and South Kuril populations separated for a long time. During this period of geographic isolation, the chromosome sets of individuals from the western Sea of Japan and the southern Kuril Islands could have formed independently of each other, despite their visual identity (cytotype V). One of the southern Kuril specimens has haplotype 1b, which connects all other haplotypes found in *M. stelleri*, and may thus be the closest to the ancestral haplotype. Contrary to the results of mtDNA analysis, the karyological data point to a significant divergence of *M. stelleri* (2n = 40) from the coastal waters off the southern Kuril Island. This discrepancy may be caused by the high rate of change in karyological traits compared to that of DNA markers (Lukhtanov and Kuznetsova 2009).

Our results do not support the assumption that the individuals of *M. stelleri* from the Sea of Japan and the Sea of Okhotsk belong to different species (Podlesnykh and Moreva 2014). Judging by the values of genetic and chromosomal polymorphism, the low level of genetic differentiation, and the frequency and spatial distribution of haplotypes, this species is relatively young. It can be suggested that the fragmentation of the single range of *M. stelleri*, which took place during the last Quaternary glaciation 20–25 thousand years ago, resulted in the geographic isolation of populations from the Sea of Japan and Sea of Okhotsk. The accumulation of Robertsonian translocations in *M. stelleri* from the western Sea of Japan presumably occurred during this period. We suggest that, as the glaciation ended, the ocean transgression and restoration of connections between the seas of the Northwest Pacific (Nishimura 1964; Lindberg 1972; Yokoyama et al. 2007; Matsuzaki et al. 2018) allowed the secondary colonization of *M. stelleri*, which likely resulted in a significant chromosomal polymorphism in the northern Sea of Japan. The paleoclimate events also influenced the distribution of genetic variation in *M. stelleri*. The formation of the three phylogenetic groups is likely associated with the geographic isolation of ancestral forms in different parts of the species range, and with the limited gene exchange and secondary contact because of migrations after the last ice age (Briggs 2003; Altukhov 2005).

## Conclusions

Our data have revealed a similarity in chromosome sets, as well as low levels of differentiation in mtDNA between *M. stelleri* from the Sea of Okhotsk, the Sea of Japan, and the coast of Shikotan Island (southern Kuril Islands), thus, confirming that these represent a single, yet variable, species across its geographic range. The significant chromosomal polymorphism and the presence of common haplotypes in the studied samples indicate their recent origin from a common ancestor and/or relatively recent contacts within the range. The contribution of different Robertsonian translocations to the formation of cytotypes (II–IV and V) of individuals from the northern and western Sea of Japan allows us to conclude that they diverged from the Sea of Okhotsk *M. stelleri* independently of each other. The star-shaped structure of the haplotype network with a central ancestral haplotype indicates a connection between all constituent haplotypes and the ancestral position of the southern Kuril Islands haplotype (1b). The discrepancy in assessments of the divergence of individuals from the Sea of Okhotsk and waters off the southern Kuril Islands can be attributed to the different mechanisms of inheritance and rates of evolution of karyological traits and mtDNA markers.

The results of our study demonstrate the necessity of further detailed analysis of the widely sampled *M. stelleri* populations from the Pacific part of the species range and from the southern Sea of Okhotsk. Such studies should include differential chromosome staining and SNP markers, as well as comparative morphological and osteological analyses using up-to-date methods.
